# Characterization of mercury bioremediation by transgenic bacteria expressing metallothionein and polyphosphate kinase

**DOI:** 10.1186/1472-6750-11-82

**Published:** 2011-08-12

**Authors:** Oscar N Ruiz, Derry Alvarez, Gloriene Gonzalez-Ruiz, Cesar Torres

**Affiliations:** 1 Inter American University of Puerto Rico, Department of Natural Sciences and Mathematics, 500 Dr. John Will Harris, Bayamon, 00957, Puerto Rico

## Abstract

**Background:**

The use of transgenic bacteria has been proposed as a suitable alternative for mercury remediation. Ideally, mercury would be sequestered by metal-scavenging agents inside transgenic bacteria for subsequent retrieval. So far, this approach has produced limited protection and accumulation. We report here the development of a transgenic system that effectively expresses metallothionein (*mt-1*) and polyphosphate kinase (*ppk*) genes in bacteria in order to provide high mercury resistance and accumulation.

**Results:**

In this study, bacterial transformation with transcriptional and translational enhanced vectors designed for the expression of metallothionein and polyphosphate kinase provided high transgene transcript levels independent of the gene being expressed. Expression of polyphosphate kinase and metallothionein in transgenic bacteria provided high resistance to mercury, up to 80 μM and 120 μM, respectively. Here we show for the first time that metallothionein can be efficiently expressed in bacteria without being fused to a carrier protein to enhance mercury bioremediation. Cold vapor atomic absorption spectrometry analyzes revealed that the *mt-1 *transgenic bacteria accumulated up to 100.2 ± 17.6 μM of mercury from media containing 120 μM Hg. The extent of mercury remediation was such that the contaminated media remediated by the *mt-1 *transgenic bacteria supported the growth of untransformed bacteria. Cell aggregation, precipitation and color changes were visually observed in *mt-1 *and *ppk *transgenic bacteria when these cells were grown in high mercury concentrations.

**Conclusion:**

The transgenic bacterial system described in this study presents a viable technology for mercury bioremediation from liquid matrices because it provides high mercury resistance and accumulation while inhibiting elemental mercury volatilization. This is the first report that shows that metallothionein expression provides mercury resistance and accumulation in recombinant bacteria. The high accumulation of mercury in the transgenic cells could present the possibility of retrieving the accumulated mercury for further industrial applications.

## Background

Bioremediation presents a potentially low cost and environmentally agreeable alternative to current physico-chemical remediation strategies. However, heavy metals such as mercury cannot be converted into non-toxic forms by naturally occurring bacteria. Annual global emissions estimates for mercury released into the environment are in the thousands of tons per year [1,2] while the remediation cost is in the thousands of dollars per pound. Finding new bioremediation technologies is an urgent need. Mercury is released into the environment as a result of human activities and natural events. Ionic and metallic forms of mercury can accumulate in sediments where they can be converted into highly toxic methyl mercury by bacteria. Further biomagnification of mercury through trophic levels can lead to human poisoning through seafood consumption [3].

Genetic engineering can be used to integrate genes into bacteria to enhance mercury resistance and accumulation. A method for mercury bioremediation based on the expression of the bacterial *mer *genes has been developed [4]. In this approach, mercuric ion reductase reduces ionic mercury (Hg^2+^) to elemental mercury (Hg^0^), which is then volatilized from the cell. The disadvantage of this approach is that elemental mercury is released into the environment where it accumulates and can eventually be converted into very toxic organomercurials.

Metallothionein and polyphosphates are heavy metal scavenging molecules that have been expressed in bacteria with the purpose of increasing heavy metal resistance and accumulation. Metallothioneins are cystein rich, low molecular weight metal-binding proteins encoded by the *mt *genes that can sequester metal ions in a biologically inactive form [5,6]. Polyphosphates are negatively charged polymers of orthophosphates that can bind metal ions [7]. The *ppk *gene encodes the enzyme polyphosphate kinase, which is the responsible for polyphosphates biosynthesis in bacteria.

Attempts have been made to express metallothioneins and polyphosphates in bacteria. However, bacterial expression of metallothionein (MT) was shown to be unstable [8] and had to be fused with glutathione-S-transferase (GST) [9]. Explanations for the instability of metallothionein in bacteria included: rapid degradation of the transcripts and small peptide, low protein expression, and interference with redox pathways [10,11]. Despite the high levels of GST-MT fusion protein shown in previous reports, the transgenic bacteria failed to grow in mercury concentrations above 5 μM [9,12-16]. Usually, a 5 μM mercury concentration is considered nonlethal to untransformed bacteria. It has been reported that GST may have a role in mercury detoxification [17-19]. Using GST as a carrier for MT may complicate evaluating the characteristics of MT as a mercury bioremediation agent in transgenic bacteria. It is safe to say that metallothionein has not provided adequate resistance to mercury as of yet [20].

Other research groups have focused their efforts on the expression of the polyphosphate kinase (*ppk*) gene in transgenic bacteria to increase the levels of polyphosphates and mercury resistance [21,22]. Transgenic bacteria expressing *ppk *was shown to withstand and accumulate up to 16 μM of mercury from solutions [21,22]. Others reports indicated that both polyphosphate kinase and polyphosphatase enzymes are needed in order to obtain heavy metal resistance [23-25].

The low levels of mercury resistance achieved by engineered bacteria in previous reports preclude their application as an effective bioremediation system. It was our goal to develop a genetically engineered bacterial system capable of providing high expression of metallothionein and polyphosphate kinase to promote effective mercury bioremediation. We also compared the bioremediation efficiency of transgenic bacteria expressing metallothionien and polyphosphate kinase to understand which of these genes is best suited for mercury bioremediation. Finally, we characterized the bioremediation efficiency of the metallothionein-expressing bacteria.

## Methods

### Quantification of Transgene Expression

Total cellular RNA was isolated using the RNeasy Mini Kit (Qiagen, Germantown, MD) from 1 ml of transformed and untransformed *E. coli *JM109 grown in Luria Bertani (LB) broth for 16 hours at 37°C and 300 rpm agitation. The RNA samples were treated with DNAse I at a concentration of 100 μg/mL to remove any residual DNA, normalized, and then reverse transcribed employing the random primers protocol of the AccuScript cDNA Kit (Stratagene, La Jolla, CA). The cDNA was analyzed by quantitative real-time PCR using the MJ MiniOpticon real-time PCR system (BioRad, Herculex, CA) with a two-step amplification program with post-amplification melt curve analysis. Gene-specific qPCR primers and synthetic oligonucleotide standards were developed. The *mt-1 *and *ppk *synthetic oligonucleotides spanned the region covered by the *mt-1 *and *ppk *qPCR primers. The synthetic oligonucleotides were diluted from 1 × 10^7 ^copies/μl to 1 × 10^2 ^copies/μl to produce the qPCR quantification standards. In order to differentiate the introduced *ppk *gene from the endogenous *ppk *gene in the bacteria through real-time PCR, the forward primer was designed to anneal upstream of the introduced *ppk *gene start codon within the g10 region. The reverse reaction primer annealed within the *ppk *gene. Only the introduced *ppk *gene contains the g10 region upstream and can be detected from cDNA samples with this primer combination.

### Mercury Resistance Bioassay

Bacterial clones pBSK-P16S-g10-*mt1*-rpsT, pBSK-P16S-g10-*ppk-*rpsT, pBSK-g10-*mt1*-rpsT, pBSK-g10-*ppk-*rpsT, and untransformed *E. coli *JM109 grown for 16 hours in sterile Luria Bertani (LB) broth at 37°C with 300 rpm agitation were used as inoculums for the mercury resistance bioassays. The bacterial clones described above and the untransformed *E. coli *were inoculated in triplicate to an initial concentration of 0.01 OD_600 _in 5 ml of LB broth amended with HgCl_2 _to final concentrations of 0, 5, 10, 20, 40, 80, 100, 120, 140, and 160 μM. The lac promoter in the pBlueScript vector was induced by the addition of 200 μg/ml Isopropyl β-D-1-thiogalactopyranoside (IPTG) to the culture medium. The culture tubes were incubated at 37°C with 300 rpm agitation for a period of 16 and 120 hours. The absorbance of the cultures was measured at 600 nm.

### Mercury Accumulation Quantification

Bacteria cell pellets were obtained by centrifugation from 5 ml of pBSK-P16S-g10-*mt1*-rpsT and untransformed bacteria cultures grown for 72 and 120 hour in the presence of 120 μM HgCl_2_. The cell pellets were washed three times with fresh LB medium and then acid-digested by stepwise additions of 70% (v/v) nitric acid, 30% (v/v) hydrogen peroxide, and concentrated HCl at 95°C adapting EPA method 3010A [26]. Reagent blanks and spiked control samples were treated as described.

NIST traceable Mercury (Hg) PerkinElmer Pure Calibration Standard 1000 ppm (Lot #14-04HG; PE # N9300133; CAS # HG7439-97-6) was used to produce the quantification standards and spike controls. Matrix spiked controls were produced by adding 100 ng/ml Hg to *E. coli *cell pellets recovered by centrifugation from 5 ml LB cultures that were grown for 16 hours without mercury. The average recovery value for the matrix spiked controls was 96.7 ± 4.68 ng/ml or a 96.7%. A characteristic concentration check was performed to determine instrument calibration. A check standard of concentration different to the curve standards was used to confirm the calibration. Two method blanks were run per extraction batch for quality control. The limit of detection for the cold vapor atomic absorption spectroscopy (CVAAS) analysis was 15 ng/ml. All samples were analyzed in triplicates using an AAnalyst 200 Perkin Elmer Spectrometer with a MHS-15 Mercury-Hydride System. The mercury accumulation in μM was calculated by multiplying the ng/ml (μg/l) value obtained from the instrument by the appropriate dilution factor used to keep the sample within the standard curve range, and then divided by the molecular mass of mercury (200.59 μg) in a μmol.

## Results and Discussion

### Construction of Enhanced Expression Vectors for Bioremediation

Limited mercury resistance and accumulation has been reported in transgenic bacteria expressing the *mt *and *ppk *genes. To overcome previous problems, we developed an expression construct optimized for the transcription, translation, and mRNA stability of the transgenes. Transcription optimization was achieved by using a strong constitutive promoter derived from the tobacco plastid 16S ribosomal RNA gene (P16S). The 16S *rrn *gene is one of the most transcribed genes in the bacterial cell [27-29]. The plastid P16S promoter has proven to be functional in multiple bacteria species [29]. Transcript termination and post-transcriptional transcript stability was obtained by the insertion of the rpsT terminator element. The rpsT element was derived from the 3' untranslated region (UTR) of the plastid *rps *16 gene. This terminator element was placed downstream from the transgene termination codon. The 3'UTR element enhances transcript stability by forming a secondary structure at the 3' end of the mRNA [30]. A 5'UTR element obtained from bacteriophage T7 gene 10 was placed upstream of the transgene initiation codon in order to enhance translation [31]. The gene 10 5'UTR, also known as g10, is a heterologous transcriptional enhancer element that acts as an efficient ribosome binding site in bacteria.

The mouse *mt-1 *gene, which codes for metallothionein-1, and the *Escherichia coli *(*E. coli*) *ppk *gene, which produces the enzyme polyphosphate kinase, were both obtained by polymerase chain reaction (PCR) amplification using gene-specific primers. The plasmid pCMV-SPORT10, which contains the mouse *mt-1 *cDNA, and *E. coli *genomic DNA carrying the *ppk *gene, were used as DNA templates for PCR. The gene-specific forward primers were engineered to include the g10 element sequence while the reverse primers had the rpsT element. Both PCR amplicons were cloned into the commercially available pBlueScript (pBSK) vector in-frame to the vector's inducible lac promoter to produce the pBSK-g10-mt1-rpsT and pBSK-g10-ppk-rpsT vectors. The lac promoter was considered a weak promoter [32].

The expression constructs containing the 16S promoter (P16S) were developed by PCR amplification of the g10-mt1-rpsT and g10-ppk-rpsT cassettes with a g10-specific forward primer that contained the P16S sequence upstream of the g10 region. The reverse reaction primers were the same primers used in the initial amplification of *mt-1 *and *ppk *genes. The P16S-g10-mt1-rpsT and P16S-g10-ppk-rpsT amplicons were cloned into the pBSK vector to form the final expression vectors. All vectors were transformed into *E. coli *strain JM109.

### Transgene Expression Analysis

**Figure 1:**
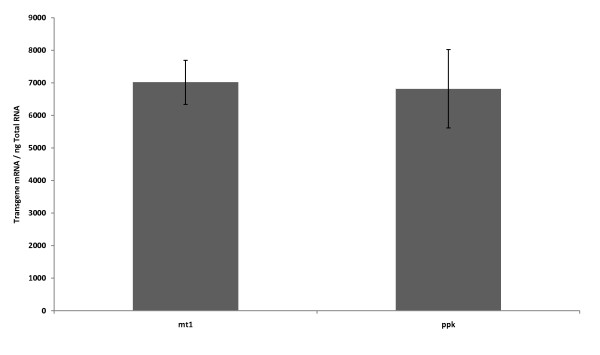
**Transgene expression analysis**. Quantitative RT-PCR analysis was performed on equal amounts of RNA extracted from transgenic *E. coli *expressing the *mt-1 *(A) and *ppk *(B) genes, and untransformed *E. coli *(wt). (n = 3).

Total RNA samples extracted from the pBSK-P16S-mt1-rpsT and pBSK-P16S-g10-ppk-rpsT bacterial clones were reverse transcribed and analyzed by quantitative real-time PCR (Figure 1). The results indicated that the levels of *mt-1 *and *ppk *mRNA were very similar in both transgenic bacteria, with 7,016 and 6,819 transgene copies per ng of total RNA, respectively (Figure 1). Control experiments using cDNA from untransformed *E. coli *showed no expression of the transgenes. These results indicated that the expression constructs provided abundant transcription and similar mRNA levels independent of the transgene being expressed. Contrary to previous reports that indicated that *mt *expression was unstable due to rapid degradation of transcripts [9-11], we have shown that *mt-1 *transcripts containing the rpsT are stable. Transcript abundance is an important factor that regulates the amount of protein produced in bacteria. High levels of transgene mRNA usually correlate with high protein abundance.

In bacteria, gene expression is often regulated at the transcriptional level. However, improvement in translation can still be achieved by the use of heterologous ribosome binding site elements such as the g10. Codon bias has been singled out as another factor that may influence protein expression in bacteria. However, *E. coli *is a bacteria with a neutral GC content, which makes it more amenable to the expression of eukaryotic proteins, such as metallothionein, which is about 60% GC. Codon bias has recently been identified as an important factor affecting the translation of longer genes in bacteria; however this effect was less significant in smaller genes of less than 500 bp [33]. It is possible that codon bias was not affecting *mt-1 *translation because of its small size (221 bp).

### Mercury Resistance Bioassays

Bacterial clones harboring the plasmids pBSK-g10-mt1-rpsT, pBSK-P16S-mt1-rpsT, pBSK-g10-ppk-rpsT, pBSK-P16S-g10-ppk-rpsT, and untransformed *E. coli *JM109 were grown in Luria Bertani (LB) broth in the presence of HgCl_2 _(Hg) at concentrations of 0, 5, 10, 20, 40, 80, 100, 120, 140, and 160 μM. Untransformed (wild type) *E. coli *was used as a negative control in these assays. The absorbance was measured at 600_nm _for each bacterial clone after 16 and 120 hours of incubation in order to determine growth and their relative resistance to mercury.

**Figure 2:**
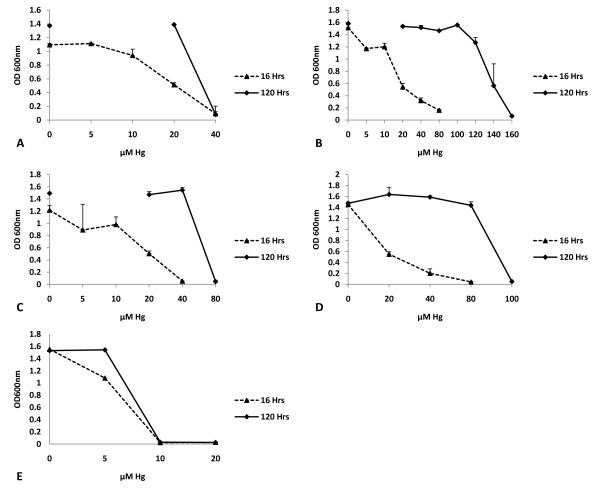
**Mercury resistance bioassay**. Bacterial clones pBSK-g10-mt1-rpsT (A), pBSK-P16S-g10-mt1-rpsT (B), pBSK-g10-ppk-rpsT (C), pBSK-P16S-g10-ppk-rpsT (D), and untransformed *E. coli *(E) were grown in LB media with 0, 5, 10, 20, 40, 80, 100, 120, 140, and 160 μM of HgCl_2_. Bacterial growth was established by measuring the absorbance at 600 nm after 16 and 120 hours. (n ≥3).

The results showed that wild type *E. coli *cells can only withstand concentrations of 5 μM Hg, which are considered nonlethal (Figure 2E). Even at this concentration, the growth rate was reduced over the 0 μM Hg culture. At 10 μM Hg and above, complete cell inhibition was observed at 16 and 120 hours (Figure 2E). A very different result was observed for the transgenic clones.

The pBSK-g10-mt1-rpsT bacterial clone showed good resistance up to 20 μM Hg after 16 hours of incubation. However, growth was reduced when compared with the 0 μM Hg sample (Figure 2A). After 120 hours of incubation, the pBSK-g10-mt1-rpsT clone was able to achieve a saturation level similar to the 0 μM sample (Figure 2A). This vector did not provide resistance to concentrations of 40 μM Hg or more. A similar study performed with the pBSK-P16S-g10-mt1-rpsT clone showed that this bacteria grew in concentrations of up to 80 μM Hg in 16 hours. Nevertheless, some growth reduction was observed after the 10 μM concentration (Figure 2B). The pBSK-P16S-g10-mt1-rpsT bacteria grew effectively in concentrations of up to 120 μM Hg when incubated for 120 hours, achieving growth levels equal to samples without Hg in concentrations as high as 100 μM Hg. Only at the 120 μM Hg concentration was a slight growth reduction perceived (Figure 2B). The pBSK-P16S-g10-mt1-rpsT bacteria was even able to grow at 140 μM Hg, though to a more limited extent. The resistance levels achieved by the pBSK-P16S-g10-mt1-rpsT bacteria were about 12-times better than those reported for transgenic bacteria expressing MT-GST fusion [9,12-16]. These results indicated that by using a combination of transcriptional and translational enhancer elements, the *mt-1 *gene can be effectively expressed to provide maximum protection against the toxic effects of Hg. Furthermore, we demonstrated that the use of the right promoter and regulatory elements combination is key in effective mercury resistance. As observed, the pBSK-P16S-g10-mt1-rpsT transgenic bacteria that uses the constitute 16S *rrn *promoter was at least 6-times more resistant that the pBSK-g10-mt1-rpsT transgenic clone, which is regulated by the weak lac promoter.

When the pBSK-g10-ppk-rpsT bacterial clone was grown for 16 hours it was able to grow in the presence of 20 μM Hg (Figure 2C). However, the pBSK-g10-ppk-rpsT bacteria grew saturation at 20 and 40 μM Hg (Figure 2C) after a 120 hour incubation period. Both the pBSK-g10-ppk-rpsT and pBSK-g10-mt1-rpsT clones grew in 20 μM Hg when incubated for 16 hours. However, after 120 hours, the pBSK-g10-ppk-rpsT clone had better resistance than the pBSK-g10-mt1-rpsT clone; achieving growth saturation in 40 μM Hg (Figure 2A and 2C).

Mercury bioassays performed with the pBSK-P16S-g10-ppk-rpsT bacteria revealed that this transgenic bacteria was able to grow in Hg concentrations of up to 40 and 80 μM after 16 and 120 hours of incubation, respectively (Figure 2D). This level of resistance is 5 times higher than previously reported for bacterial cells expressing the *ppk *gene [21,22]. These results clearly demonstrate that the use of the constitutive P16S promoter is important for maximum protection against mercury.

It has been shown that transgenic bacteria expressing *ppk *has higher polyphosphate levels and higher mercury resistance than untransformed bacteria [21,22]. Others have reported that the polyphosphatase encoded by the *ppx *gene is required along with the *ppk *gene to protect the cell from the toxic effects of heavy metals [23-25]. While we did not genetically engineer polyphosphatase in our transgenic bacteria, it is possible that endogenous polyphosphatase is completing the polyphosphate pathway in *ppk *transgenic bacteria. More studies are needed to elucidate the role of *ppk *and *ppx *in polyphosphate-mediated heavy metal resistance.

Although the pBSK-P16S-g10-ppk-rpsT and pBSK-P16S-g10-mt1-rpsT bacteria had very similar mRNA levels, the *mt-1 *transgenic bacteria was 1.8-times more resistant to mercury than the *ppk *transgenic bacteria (Figure 2). A possible explanation for this is that the cell is modulating the production of polyphosphates by restricting the availability of ATP in order to prevent the depletion of the cellular ATP pool. It is likely that there was not enough endogenous polyphosphatase to complete the polyphosphate metabolic pathway given that the *ppx *gene was not genetically engineered along with the *ppk *gene. Simultaneous expression of *ppk *and *ppx *could possibly lead to improved resistance in the future.

### Mercury Bioremediation Assay

A study was designed to determine the bioremediation capabilities of the pBSK-P16S-g10-mt1-rpsT bacteria clone. The *mt-1 *transgenic bacteria was chosen over the *ppk *transgenic bacteria for further study because it provided the highest resistance against mercury. Therefore, the *mt-1 *bacteria presents the greatest potential for mercury bioremediation. This is the first time that metallothionein has been show to protect bacteria against the harmful effects of mercury and because of this it is important to demonstrate that metallothionein can also provide mercury bioremediation capabilities to the transgenic bacteria. In the case of *ppk*, Pan-Hou et al., [21,22] had demonstrated that recombinant *E. coli *expressing the *ppk *gene can accumulate up to 16 μM of mercury. While the level of mercury accumulation was low, it was demonstrated that expression of *ppk *in transgenic bacteria increased mercury accumulation.

**Figure 3:**
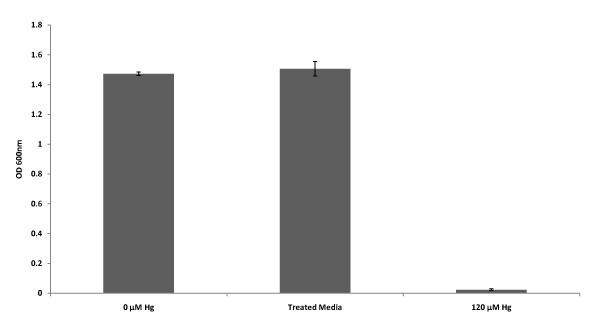
**Mercury bioremediation assay**. Growth of untransformed *E. coli *bacteria in media without HgCl_2_, with 120 μM HgCl_2_, and in treated medium was measured after a 16 hours culture period at 37°C. The untransformed bacteria was inoculated to an initial absorbance of 0.01. Treated medium was LB culture media that was initially amended with 120 μM HgCl_2_, inoculated with *mt-1 *transgenic bacteria, and allowed to grow for 120 hours. After the 120 hours, the *mt-1 *transgenic bacteria was removed from the LB media by centrifugation and filter sterilization. Growth was determined by measuring absorbance at 600 nm.

Here, untransformed *E. coli *JM109 cells were inoculated to an absorbance of 0.01 in LB medium without mercury, LB medium with 120 μM HgCl_2 _(Hg), and treated LB medium. The treated LB medium was produced by growing the pBSK-P16S-g10-mt1-rpsT bacteria clone in LB medium containing 120 μM Hg for 120 hours. After 120 hours incubation, the *mt-1 *bacteria were removed from the liquid medium by centrifugation at 13,000 rpm for 2 minutes and the supernatant was collected and filter sterilized by using a 0.22 μm filter to remove any residual transgenic cells lingering from the previous inoculation. The sterile treated LB medium was re-inoculated with untransformed *E. coli *at an absorbance of 0.01 and grown for 16 hours. A growth control reaction was produced by inoculating *E. coli *into LB medium containing 120 μM Hg that was centrifuged and passed through a 0.22 μm filter. The purpose of this process was to mimic the treatment given to the treated medium, and to account for any Hg loss due to the centrifugation or filtration. The results showed that untransformed *E. coli *grew to saturation in medium without mercury and in the treated medium after 16 hours of incubation (Figure 3). Untransformed *E. coli *failed to grow in medium containing 120 μM Hg (Figure 3). These results demonstrated that metallothionein expression not only provided resistance to mercury, but also enhanced mercury removal from liquid media to an extent that allows normal growth of untransformed *E. coli*. We inferred that the concentration of mercury left in the treated medium was less than 5 μM because untransformed *E. coli *was able to grow to saturation in a 16 hours period (Figure 2E). A sterility check control reaction that was undertaken to demonstrate that *mt-1 *transgenic cells were not found in the treated media was done by incubating 1 ml of treated medium for 16 hours and then measuring the absorbance of the broth. The results showed no bacterial growth and zero absorbance.

Finally, to demonstrate that the pBSK-P16S-g10-mt1-rpsT bacteria was indeed accumulating mercury, bacteria cell pellets obtained from 5 mL LB cultures containing 120 μM Hg after 72 and 120 hours of growth were analyzed by cold vapor atomic absorption spectrometry (CVAAS). The results showed that the pBSK-P16S-g10-*mt1*-rpsT bacteria was very efficient at uptaking Hg; accumulating 51.6 ± 14.1 μM Hg in the first 72 hours and 100.2 ± 17.6 μM Hg by 120 hours. The increment in Hg accumulation observed at 120 hours could be due to more bacterial growth and increased time for mercury translocation to the cell. These results validated our previous observations indicating that untransformed *E. coli *could grow in media that was previously bioremediated by the pBSK-P16S-g10-*mt1*-rpsT transgenic bacteria. We conclude that the *mt-1 *transgenic bacteria was capable of bioremediating and accumulating mercury from contaminated liquids.

### Visual Changes in Transgenic Bacteria under Mercury Conditions

**Figure 4:**
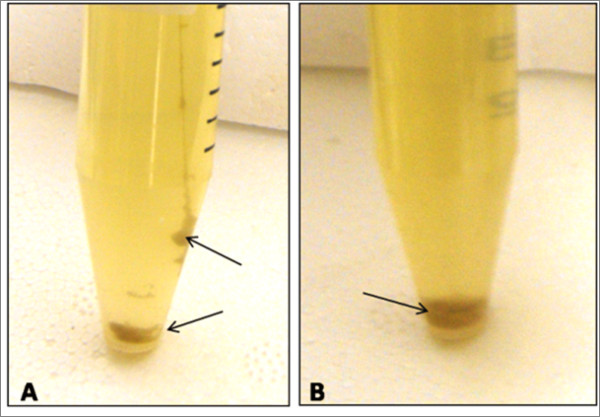
**Visual changes in transgenic bacteria under mercury conditions**. Black arrows indicate areas of aggregation, precipitation, and color change. A, pBSK-P16S-g10-ppk-rpsT bacteria at 80 μM of HgCl_2_. B, pBSK-P16S-g10-mt1-rpsT bacteria at 120 μM of HgCl_2_. Pictures were taken after 72 hours of growth.

It was also observed that the pBSK-P16S-g10-mt1-rpsT and pBSK-P16S-g10-ppk-rpsT bacterial clones formed aggregates or clumps that precipitated from the solution after enough contact time with high mercury concentrations (Figure 4A and 4B). The aggregation and precipitation effects were observed when the transgenic bacteria were grown in mercury concentrations equal or higher to 80 μM for a period of at least 24 hours (Figure 4). These effects were not observed at lower mercury concentrations. The pBSK-P16S-g10-mt1-rpsT and pBSK-P16S-g10-ppk-rpsT clones also acquired a darker color which was visible at concentrations equal or higher than 40 μM Hg (Figure 4). Since the aggregation, precipitation, and color changes were only observed when the bacterial clones were grown in high mercury concentrations, it is possible that these effects were dependent on high mercury resistance and accumulation by the transgenic bacteria. These cellular changes can potentially be used as markers to determine the progress and extent of the bioremediation process. Also, the clumping and precipitation characteristics of these transgenic bacteria can be applied to the development of a simple sifting mechanism to recover cells that have accumulated high mercury concentrations.

## Conclusion

This study describes the development of a new mercury bioremediation technology based on accumulation of mercury inside the bacterial cell. Here, we provide the first unequivocal example of metallothionein protection against mercury in bacteria. Furthermore, metallothionein has been efficiently expressed without being fused to a carrier protein, achieving high mRNA levels, mercury resistance and accumulation. Efficient expression of the mouse *mt-1 *and bacterial *ppk *genes in transgenic bacteria was achieved by using a transcriptional and translational enhanced expression vector. Transgene mRNA levels ranged from 6,819 to 7,016 copies per ng of RNA, for *ppk *and *mt-1 *genes respectively. The similar transgene expression in *mt-1 *and *ppk *transgenic bacteria indicate that it is possible to express prokaryotic and mammalian genes effectively in bacteria if the vector is engineered with proper regulatory elements to maximize expression. Furthermore, obtaining similar expression levels facilitates the comparison of the bioremediation capabilities provided by each of the transgenes.

Here we have demonstrated beyond a doubt that our *ppk *and *mt-1 *transgenic bacteria were able to grow in very high mercury concentrations up to 80 and 120 μM, respectively. Mercury bioassays indicate that the *mt-1 *and *ppk *bacteria were about 12-times and 6-times more resistant to mercury than the best literature reports for the same genes. Furthermore, results show that metallothionein provided higher mercury resistance and accumulation than polyphosphate kinase under the conditions we tested. We showed that our *mt-1 *transgenic bacteria removed mercury from liquid matrices by accumulating mercury to high concentrations. Cold vapor atomic absorption spectrometry analysis of *mt-1 *transgenic bacteria exposed to 120 μM Hg for 120 hours revealed that the bacteria was able to accumulate up to 100.2 ± 17.6 μM Hg from the liquid media. This result clearly demonstrates that the *mt-1 *transgenic bacteria remediated mercury by accumulation within the cell. The extent of mercury remediation was such that the remediated growth media supported the growth of untransformed bacteria afterwards. The high mercury resistance and accumulation by the *mt-1 *transgenic bacteria indicates that metallothionein was expressed in the active form without the need to be fused to a carrier protein to confer stability. The transgenic bacterial bioremediation system described in this study presents the first viable bioremediation technology for mercury removal from liquid matrices. The levels of resistance observed in *mt-1 *and *ppk *transgenic bacteria were equal or better than the best reports for transgenic bacteria expressing the *mer *operon. Nevertheless, our system is more suitable for mercury bioremediation because it does not volatilize elemental mercury into the atmosphere, which makes it a safer and more attractive technology for commercial application. Other characteristics of the transgenic bacterial system that may facilitate the commercial application of this system were the observed aggregation, precipitation, and color change of the transgenic bacterial cells when exposed to high mercury levels. These visual changes may be used as indicators to assess growth and mercury accumulation. More studies are needed to further understand the processes of mercury absorption, accumulation, and resistance in transgenic bacteria expressing metallothionein and polyphosphate kinase.

## Authors' contributions

ONR conceived and designed the study, wrote the manuscript, and lead in the mercury bioassays, vector construction, molecular characterization, and mercury quantification. DA carried out the mercury bioassays and participated in vector construction and molecular characterization. GG participated in vector construction and transformations. CT carried out the mercury quantifications by CVAAS. All authors read and approved the final manuscript.
